# The effect of polyphenols on DNA methylation-assessed biological age attenuation: the DIRECT PLUS randomized controlled trial

**DOI:** 10.1186/s12916-023-03067-3

**Published:** 2023-09-25

**Authors:** Anat Yaskolka Meir, Maria Keller, Anne Hoffmann, Ehud Rinott, Gal Tsaban, Alon Kaplan, Hila Zelicha, Tobias Hagemann, Uta Ceglarek, Berend Isermann, Ilan Shelef, Matthias Blüher, Michael Stumvoll, Jun Li, Sven-Bastian Haange, Beatrice Engelmann, Ulrike Rolle-Kampczyk, Martin von Bergen, Frank B. Hu, Meir J. Stampfer, Peter Kovacs, Liming Liang, Iris Shai

**Affiliations:** 1 The Health & Nutrition Innovative International Research Center, Faculty of Health Sciences, Ben-Gurion University of the Negev, P.O. Box 653, 8410501 Be’er Sheva, Israel; 2 Department of Epidemiology, Harvard T.H. Chan School of Public Health, 655 Huntington Avenue, Boston, MA 02115 USA; 3 Helmholtz Institute for Metabolic, Obesity and Vascular Research (HI-MAG) of the Helmholtz Center Munich at the University of Leipzig and University Hospital Leipzig, 04103 Leipzig, Germany; 4 Medical Department III – Endocrinology, Nephrology, Rheumatology, University of Leipzig Medical Center, University of Leipzig, Liebigstrasse 21, 04103 Leipzig, Germany; 5 Soroka University Medical Center, 84101 Be’er Sheva, Israel; 6 Institute of Laboratory Medicine, Clinical Chemistry, and Molecular Diagnostics, University of Leipzig Medical Center, 04103 Leipzig, Germany; 7 Division of Preventive Medicine, Department of Medicine, Brigham and Women’s Hospital and, Harvard Medical School, Boston, MA 02115 USA; 8 Department of Molecular Systems Biology, Helmholtz Centre for Environmental Research GmbH, 04318 Leipzig, Germany; 9 Institute of Biochemistry, Faculty of Life Sciences, University of Leipzig, 04103 Leipzig, Germany; 10 Department of Nutrition, Harvard T.H. Chan School of Public Health, Boston, MA 02115 USA; 11 Channing Division of Network Medicine, Department of Medicine, Brigham and Women’s Hospital and Harvard Medical School, Boston, MA 02115 USA; 12 Faculty of Medicine, Leipzig University, Leipzig, 04103 Germany

**Keywords:** Epigenetics, Weight loss, Green-MED diet, Urolithins, Tyrosol, Methylation age, Urine metabolomics

## Abstract

**Background:**

Epigenetic age is an estimator of biological age based on DNA methylation; its discrepancy from chronologic age warrants further investigation. We recently reported that greater polyphenol intake benefitted ectopic fats, brain function, and gut microbiota profile, corresponding with elevated urine polyphenols. The effect of polyphenol-rich dietary interventions on biological aging is yet to be determined.

**Methods:**

We calculated different biological aging epigenetic clocks of different generations (Horvath2013, Hannum2013, Li2018, Horvath skin and blood2018, PhenoAge2018, PCGrimAge2022), their corresponding age and intrinsic age accelerations, and DunedinPACE, all based on DNA methylation (Illumina EPIC array; pre-specified secondary outcome) for 256 participants with abdominal obesity or dyslipidemia, before and after the 18-month DIRECT PLUS randomized controlled trial. Three interventions were assigned: healthy dietary guidelines, a Mediterranean (MED) diet, and a polyphenol-rich, low-red/processed meat Green-MED diet. Both MED groups consumed 28 g walnuts/day (+ 440 mg/day polyphenols). The Green-MED group consumed green tea (3–4 cups/day) and Mankai (*Wolffia globosa* strain) 500-ml green shake (+ 800 mg/day polyphenols). Adherence to the Green-MED diet was assessed by questionnaire and urine polyphenols metabolomics (high-performance liquid chromatography quadrupole time of flight).

**Results:**

Baseline chronological age (51.3 ± 10.6 years) was significantly correlated with all methylation age (mAge) clocks with correlations ranging from 0.83 to 0.95; *p* < 2.2e − 16 for all. While all interventions did not differ in terms of changes between mAge clocks, greater Green-Med diet adherence was associated with a lower 18-month relative change (i.e., greater mAge attenuation) in Li and Hannum mAge (beta =  − 0.41, *p* = 0.004 and beta =  − 0.38, *p* = 0.03, respectively; multivariate models). Greater Li mAge attenuation (multivariate models adjusted for age, sex, baseline mAge, and weight loss) was mostly affected by higher intake of Mankai (beta =  − 1.8; *p* = 0.061) and green tea (beta =  − 1.57; *p* = 0.0016) and corresponded with elevated urine polyphenols: *hydroxytyrosol*, *tyrosol*, and *urolithin C* (*p* < 0.05 for all) and *urolithin A* (*p* = 0.08), highly common in green plants. Overall, participants undergoing either MED-style diet had ~ 8.9 months favorable difference between the observed and expected Li mAge at the end of the intervention (*p* = 0.02).

**Conclusions:**

This study showed that MED and green-MED diets with increased polyphenols intake, such as green tea and Mankai, are inversely associated with biological aging. To the best of our knowledge, this is the first clinical trial to indicate a potential link between polyphenol intake, urine polyphenols, and biological aging.

**Trial registration:**

ClinicalTrials.gov, NCT03020186.

**Supplementary Information:**

The online version contains supplementary material available at 10.1186/s12916-023-03067-3.

## Background

Accelerated biological aging, apart from chronological age, is associated with the pathogenesis of chronic morbidities, such as cardiovascular, musculoskeletal, renal, neurodegenerative, and neoplastic diseases [1, 2]. Biological aging can be assessed using DNA methylation [3], known as methylation age (mAge), which is highly correlated with chronological age. Higher mAge signatures (mAge and the regressed mAge of age (age acceleration)) are associated with all-cause mortality [4, 5], cardiovascular morbidity and mortality [5–8], air pollution [9], occupational exposures [10], and body mass index (BMI) [11].

A healthy lifestyle combining a balanced diet including dietary supplements and vitamins, good sleep, and stress management beneficially alters the mAge signatures [12–15]. We have recently reported that among abdominally obese participants in the CENTRAL weight-loss trial, biological aging, measured as mAge changes from pre- to post-intervention, was significantly attenuated among individuals who experienced successful weight loss and improved magnetic resonance imaging (MRI)-assessed liver fat status after 18 months of intervention [16]. Also, lower mAge and mAge residuals (i.e., age acceleration) were directly associated with lower adiposity and glycemic markers at baseline [16].

The Mediterranean (MED) diet might increase lifespan and improve aging [17] due to its unique combination of fatty acids, antioxidants, vitamins, and phytochemicals. Polyphenols, metabolites with antioxidant properties that are enriched in the MED diet, may affect epigenetic modifications via different mechanisms [18], including inhibition of DNA methyl transferase 1 (DNMT1) [18], a central enzyme catalyzing DNA methylation [19]. However, whether eating dietary patterns rich in polyphenols may affect biological aging based on DNA methylation has yet to be elucidated.

Measuring polyphenol metabolomics to use as dietary biomarkers is challenging due to several factors: the dependence of polyphenol detection on their chemical structure, the extent of their microbial biotransformation, and the amount ingested [20]. Moreover, concentrations of metabolites change significantly over time after ingestion, with highly bioavailable polyphenols peaking in the blood shortly after ingestion before being cleared into the urine during excretion [20]. Additionally, uncertainty regarding the specific food source of polyphenols in a whole-diet regime further complicates the measurement process. Yet, our previous study suggested a metabolomic-gut-clinical axis of polyphenols [21]. Together with an assessment of Mankai plant polyphenols, which revealed 200 different phenolic compounds, we showed that some of these compounds and their derivatives were present in the urine following high-polyphenol intervention. Furthermore, some urine polyphenols as *urolithin* A were differentially elevated in the groups that consumed more dietary polyphenols.

In this study, we examined the effect of a polyphenol-rich and low-red/processed meat diet (Green-MED diet) on 18-month changes in biological age, as measured by mAge using first-, second-, and third-generation epigenetic clocks. We further examined the associations between changes in mAge and dietary intake, including specific urine polyphenols, following 18 months of dietary interventions.

## Methods

### Study design

The 18-month DIRECT-PLUS (dietary intervention randomized controlled trial polypenols-unprocessed) trial (ClinicalTrials.gov ID: NCT03020186) aimed to address the residual beneficial effect of a green Mediterranean diet, richer in green plants and lower in meat, compared with other healthy lifestyle strategies. The trial was initiated in May 2017 and was conducted in an isolated workplace (Nuclear Research Center Negev (NRCN), Dimona, Israel), where a monitored lunch was provided. This workplace includes a medical department where most medical measurements and lifestyle intervention sessions were held. Of the 378 volunteers, 294 met the inclusion criteria of age > 30 years and abdominal obesity (waist circumference (WC): men > 102 cm, women > 88 cm) or dyslipidemia (triglycerides > 150 mg/dL and high-density lipoprotein cholesterol ≤ 40 mg/dL for men, ≤ 50 mg/dL for women). The exclusion criteria were an inability to partake in physical activity (PA), a serum creatinine level ≥ 2 mg/dL, disturbed liver function, a major illness that might require hospitalization, pregnancy or lactation for women, presence of active cancer or chemotherapy within the prior 3 years, participation in another trial, treatment with warfarin (given its interaction with vitamin K), and having a pacemaker or platinum implant (due to inability to undergo magnetic resonance imaging).

All subjects gave informed consent. The protocol was approved by the Medical Ethics Board and Institutional Review Board at Soroka University Medical Centre, Be’er Sheva, Israel (0280–16-SOR). Participants received no financial compensation.

### Randomization and intervention

Randomization and intervention were described elsewhere [22]. Briefly, participants were randomly assigned to one of three intervention groups, all combined with PA recommendations (along with free gym membership):

- *Healthy dietary guidelines (HDG) group*: These participants received basic health-promoting guidelines for achieving a healthy diet.
- *MED group*: This group was instructed to adopt a calorie-restricted Mediterranean diet as described in our previous trials: DIRECT [23] and CENTRAL [24], supplemented with 28 g/day of walnuts (containing 440 mg polyphenols/day; gallic acid equivalents (GAE)) [25].
- *Green-MED group*: Besides PA and the provision of 28 g/day of walnuts, the Green-MED diet was restricted in processed and red meat and richer in plants and polyphenols. The participants were guided to consume further the following items: 3–4 cups/day of green tea and 500 ml of Mankai (*Wolffia globosa* duckweed cultivar) plant as frozen cubes. Both green tea and Mankai provided an additional daily intake of 800 mg polyphenols (GAE), according to PhenolExplorer and Eurofins lab analysis, beyond the polyphenol content in the prescribed MED diet. The MED and Green-MED diets were equally calorie-restricted (1500–1800 kcal/day for men and 1200–1400 kcal/day for women). The lifestyle interventions and motivation techniques are described in detail elsewhere [26]. All the above polyphenol food sources (green Mankai duckweed, green tea, and walnuts) were provided free of charge.

### Blood and urine samples

Blood and urine samples were taken at 8:00 AM after a 12-h fast, at baseline, and after 18 months of intervention. The blood samples were centrifuged and stored at − 80 °C until DNA isolation using the NucleoSpin Blood L, Midi kit (Macherey–Nagel) according to the manufacturer’s instructions. Body weight was measured without shoes to the nearest 0.1 kg.

### Dietary adherence assessment

We used a self-reported Food Frequency Questionnaire (FFQ) administered through a computer at baseline and after 18 months [27, 28] to assess adherence to the diet. Frequencies and portions of each food item were converted to the average daily intake for each participant. Average daily energy intake was calculated by multiplying each item’s consumption frequency by its caloric content and summing it across all foods. Using the FFQ data, we applied a Green-MED adherence score (GMD score), as previously published [29]. Briefly, the GMD score was based on the intake of nine items: walnuts, vegetables, processed meat, red meat, legumes, fruits, fish, green tea, and green Mankai. Each component’s daily intake was normalized to the average daily intake (component intake in grams/total calories). For beneficial components (vegetables, legumes, fruit, walnuts, fish, green tea, and green Mankai), individuals whose consumption was below the median were assigned a value of 0, and those whose consumption was at or above the median were assigned a value of 1. For red/processed meat intake, participants whose consumption was below the median were assigned a value of 1, and those at or above the median were assigned a value of 0. The final GMD score ranged from 0 (minimal adherence) to 9 (perfect adherence).

Urine polyphenols were measured at the Helmholtz Center for Environmental Research as described previously [21] using high-performance liquid chromatography quadrupole time of flight (HPLC-QToF) analysis (Agilent Technologies; 6540 UHD Accurate-Mass Q-ToF LC/MS instrument; Santa Clara, CA, USA). This report focused on specific 11 urine polyphenols and their derivatives highlighted in our previous publications [21, 30, 31]: urolithins (*urolithin A* and *urolithin C*) and tyrosols (including *hydroxytyrosol*). These urine polyphenols and their derivatives are presented as relative to baseline intensities (area under the curve).

### Genome-wide DNA methylation data processing and normalization

**Fig. 1:**
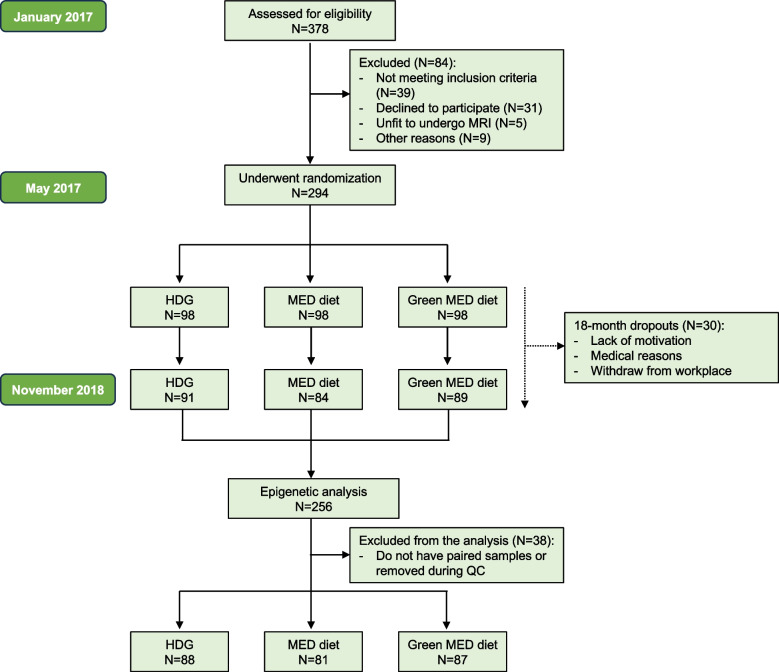
A flow diagram of the DIRECT PLUS epigenetic study. The first and last participants were enrolled in January and May 2017, respectively. HDG, healthy dietary guidelines; MED, Mediterranean

DNA methylation profiling in the blood and quality control steps were described elsewhere [32]. Briefly, blood DNA methylation was assayed at baseline (pre-intervention) and after 18 months (post-intervention); 500 ng of genomic DNA was bisulfite converted (EZ DNA Methylation Gold Kit; Zymo Research, The Netherlands), quality controlled, amplified, and hybridized on Illumina HumanMethylation850 Bead Chips (Illumina, Inc., San Diego, CA, USA). For quantification of genome-wide DNA methylation at 850 K CpG sites per sample, the Illumina iScan array scanner was used (GenomeScan, Leiden, The Netherlands). Sample-level quality control (QC) was performed using the QC report of the minfi R package (v1.38.0). Samples that did not pass QC and their paired sample were removed. This resulted in 512 paired samples (a total of 256 methylation profiles for each intervention time point for a per-protocol analysis) (Fig. 1). For the current analysis, we performed quantile normalization using the minfi R package for the 512 eligible samples. The quantile-normalized CpG sites (*N* = 865,859) without further probe filtering were used to get the beta values and to further compute all mAge estimates to minimize the number of missing CpGs that may occur in case of further removing probes that are used in the different mAge predictions. The estimated cell type was calculated using the wateRmelon R package [33].

### Methylation age assessment

We used several DNA methylation clocks in our study: (i) the age prediction formula by Li et al. [10] based on the methylation levels at 239 specific CpG sites (240 coefficients including the intercept coefficient), developed using whole blood samples and validated in multiple populations, including in our previous publication among abdominally obese White population [16], similar to the DIRECT PLUS characteristics. Of the CpGs in the Li formula, 217 were available in our data (9.21% missing). (ii) Horvath’s mAge prediction formula based on 353 CpGs [3], with 334 CpGs available (5.4% missing); (iii) Horvath skin and blood [34], with all 391 CpGs available (0% missing); (iv) Hannum’s mAge prediction [35], with 65 of 71 CpGs available (8.5% missing); (v) Levine PhenoAge [36], with all 513 CpGs available (0% missing); and (vi) PCGrimAge [37], accounting for 78,464 CpGs using the “methylclock” [38] and dnaMethyAge [39] R packages. To examine age acceleration for the above clocks, we (i) regressed chronological age from the mAge (“methylation age residuals”) and (ii) regressed the chronological age from mAge, adjusting for estimated cell type composition (Intrinsic Epigenetic Age Acceleration (IEAA)): CD8T, CD4T, natural killer (NK), B lymphocytes (Bcell), monocytes, and neutrophils. To further assess the aging pace, we also calculated DunedinPACE [40] using 173 CpGs.

### Statistical methods

The primary aim of the DIRECT-PLUS randomized controlled trial was to explore the effects of the interventions on weight and adiposity and was previously published along with the study’s full protocol and the sample size calculation elsewhere [26, 30, 41]. This analysis reports pre-specified DNA methylation secondary outcome for an 18-month time frame. The mAge clocks and their corresponding age acceleration (the residuals from a regression model of mAge on actual age; Additional file 1: Fig. S1), IEAA, and DunedinPACE were calculated at baseline and the end of the intervention, and the significance of differences was assessed using the paired *t*-test. For the 18-month change in mAge, we calculated the mAge difference between time points and the relative difference, accounting for baseline levels of mAge. We calculated Pearson (for normally distributed continuous variables) or Spearman (for non-normally distributed or categorical variables) correlations according to the distribution of the variables. Differences between the groups were tested using the chi-square or Fisher exact test (for categorical variables) or ANOVA/Kruskal–Wallis (for continuous variables). We used regression models to examine the association between the mAge relative change and intervention groups and diet indicators adjusting for confounders such as age, sex, weight loss, and baseline mAge. Interactions for the subgroup analyses were assessed using similar linear regression models. The false discovery rate (FDR) [42] was applied to correct for multiple testing, with FDR < 0.05. Statistical analyses were performed using the R software, version 4.1.

## Results

### Baseline characteristics

Baseline characteristics across the interventions stratified by sex are presented in Table 1. No differences in these characteristics were observed within each sex strata across all groups (*p* > 0.05 for all), except for metabolic syndrome > 3 components (*p* = 0.036) and DunedinPACE (*p* = 0.042) among women only. The mean age (years) and SD of the 256 participants included in this analysis were 51.1 ± 10.5 and 53.3 ± 11.4 (men and women, respectively).

**Table 1:** Baseline characteristics across the intervention groups stratified by sex^a^

	Men (*N* = 228)				Women (*N* = 28)			
	**HDG (*N* = 78)**	**MED (*N* = 72)**	**Green-MED (*N* = 78)**	***p*** between^*^	**HDG (*N* = 10)**	**MED (*N* = 9)**	**Green-MED (*N* = 9)**	*p* between^*^
Aging measurements								
Age, years	51.0 (10.3)	51.2 (10.0)	51.0 (11.1)	0.95	55.3 (10.8)	56.0 (13.2)	49.1 (10.1)	0.43
*First generation clocks*								
Li mAge, years	64.4 (8.6)	63.5 (8.4)	63.9 (9.1)	0.72	63.7 (7.8)	69.1 (12.4)	64.6 (8.7)	0.63
Horvath mAge, years	59.3 (9.5)	58.5 (8.3)	58.3 (9.5)	0.57	61.5 (9.8)	61.9 (12.0)	55.6 (7.1)	0.32
Hannum mAge, years	66.9 (9.2)	65.4 (8.8)	65.8 (9.6)	0.64	66.7 (8.2)	69.5 (12.4)	63.1 (8.2)	0.51
Horvath skin and blood mAge, years	62.4 (9.4)	61.5 (8.7)	61.4 (9.9)	0.63	66.0 (8.9)	64.5 (12.1)	61.5 (9.32)	0.39
*Second-generation clocks*								
Levine PhenoAge, years	43.3 (9.0)	41.7 (9.2)	42.9 (11.0)	0.41	46.6 (8.8)	47.5 (12.3)	41.2 (7.7)	0.21
PCGrimAge, years	65.8 (8.2)	65.1 (7.9)	65.4 (9.0)	0.82	65.9 (7.4)	68.7 (10.9)	60.8 (7.7)	0.16
*Third-generation clocks (aging pace)*								
DunedinPACE	1.12 (0.1)	1.09 (0.1)	1.12 (0.1)	0.09	1.14 (0.1)	1.17 (0.1)	1.07 (0.1)	**0.042**
Anthropometric								
Body mass index, kg/m^2^	31.4 (3.9)	31.1 (3.8)	31.1 (3.8)	0.92	30.2 (4.0)	33.5 (4.5)	30.3 (3.8)	0.17
Waist circumference, cm	110 (9.4)	111 (9.5)	110 (8.0)	0.72	102 (7.9)	107 (8.2)	98.1 (5.9)	0.09
Lifestyle habits								
Current smoker, %	17.9%	6.9%	15.4%	0.13	20.0%	22.2%	22.2%	1
Diabetics, %	10.3%	8.3%	14.1%	0.50	0%	22.2%	0%	0.19
Fatty liver, %	60.3%	59.7%	60.3%	0.86	30%	44.4%	22.2%	0.67
Metabolic syndrome => 3, %	70.5%	54.2%	66.7%	0.09	10.0%	66.7%	22.2%	**0.036**
Fish, median intake servings/day	0.38	0.38	0.38	0.77	0.25	0.27	0.32	0.92
Meat, median intake servings/day	0.59	0.48	0.55	0.45	0.44	0.54	0.34	0.42
Fruit, median intake servings/day	2.66	2.24	2.77	0.84	3.14	3.38	3.57	0.51
Vegetables, median intake servings/day	5.57	5.67	5.47	0.64	5.69	7.79	10.8	0.16
Legumes, median intake servings/day	0.23	0.23	0.27	0.48	0.14	0.23	0.29	0.59

*HDG* health dietary guidelines, *MED* Mediterranean

^a^Values are presented as mean (SD) for continuous measurements, median of servings/day for dietary data, and percentages for lifestyle habits

^*^p values according to ANOVA/Kruskal–Wallis’s test or chi-square/Fisher exact test

### Biological aging signatures and dietary intake at baseline

Out of the nine components included in the 18-month GMD score, we assessed the non-green components of baseline fish, meat, fruit, vegetable, and legume intake (as an individual daily intake with/out adjustment for daily energy). The FFQ data were available for 230 participants (89.9%) with calculated mAge. The baseline daily intake (serving/day) of fish, meat, fruit, vegetables, and legumes across groups and by sex is presented in Table 1. We further examined the correlations between the energy-adjusted daily intake (the reported intake of the dietary component divided by the daily energy estimation) of the different food components with all mAge clocks and age acceleration, including IEAA and DunedinPACE (Additional file 1: Table S1). The energy-adjusted fish intake was correlated with age (*r* = 0.16, *p* = 0.01) and all six mAge clocks (correlation ranged from 0.131 for Hannum mAge to *r* = 0.167 for Horvath mAge, *p* < 0.05 for all clocks), but not with any of the age acceleration measurements. A similar trend was observed for the energy-adjusted fruit, legume, and vegetable intakes across all clocks (Additional file 1: Table S1), except for vegetable-adjusted intake that was inversely correlated with the PCGrimAge (*r* =  − 0.141, *p* = 0.024). Meat intake was not correlated with either age or any of the mAge signatures (i.e., mAge, age acceleration, IEAA, and DunedinPACE; Additional file 1: Table S1).

### Pre- and post-intervention epigenetic clocks

**Fig. 2:**
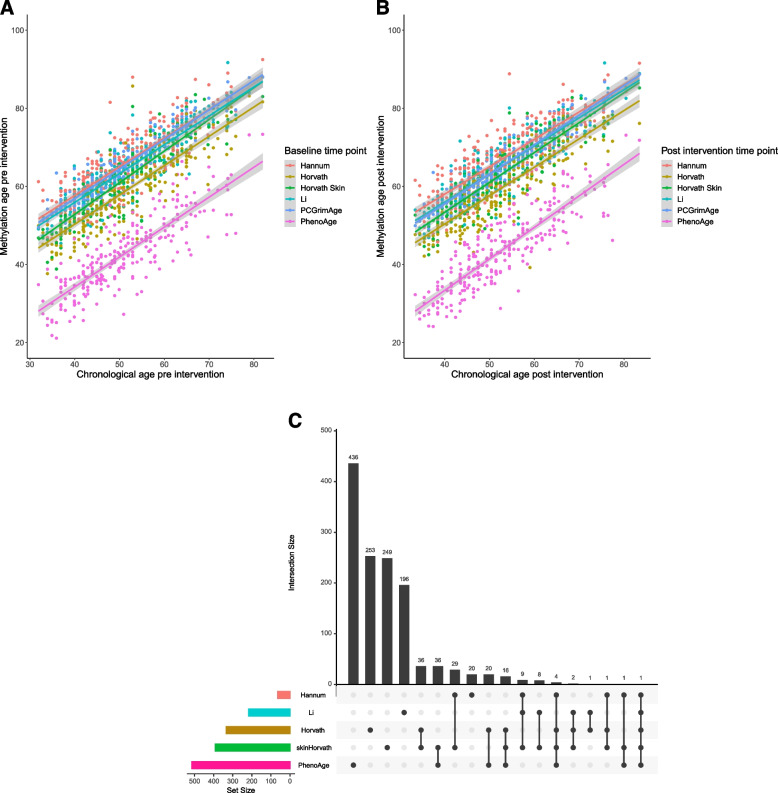
**a**–**c** Aging clocks pre- and post-intervention. **a** Correlation between age and different mAge clocks at baseline. **b** Correlation between age and different mAge clocks post-intervention. **c** Overlap between available CpGs for each clock (5 selected clocks)

Baseline chronological age (mean ± SD: 51.3 ± 10.6 years) was significantly correlated with all clocks (Fig. 2a): Li mAge (64.3 ± 8.8 years; *r* = 0.88, 95% CI [0.85, 0.91], *p* < 2.2e − 16), Horvath mAge (58.8 ± 9.2; *r* = 0.86 [0.83, 0.89], *p* < 2.2e − 16), Horvath skin and blood (62.0 ± 9.4; *r* = 0.91 [0.88, 0.93], *p* < 2.2e − 16), Hannum mAge (66.0 ± 9.2; *r* = 0.84 [0.80, 0.87], *p* < 2.2e − 16), PhenoAge (42.9 ± 9.8; *r* = 0.83 [0.79, 0.86], *p* < 2.2e − 16), and PCGrimAge (65.44 ± 8.4; *r* = 0.95 [0.94, 0.96], *p* ≪ 2.2e − 16). Similarly, at the post-intervention time point, strong correlations were observed between chronological age and the different mAge clocks (Fig. 2b; Li mAge: *r* = 0.88, 95% CI [0.86, 0.91], *p* < 2.2e − 16; Horvath mAge: *r* = 0.86 [0.83, 0.89], *p* < 2.2e − 16; Horvath skin and blood mAge: *r* = 0.90 [0.87, 0.92], *p* < 2.2e − 16; Hannum mAge: *r* = 0.83 [0.79, 0.87], *p* < 2.2e − 16; PhenoAge: *r* = 0.85 [0.82, 0.88], *p* < 2.2e − 16) and PCGrimAge (65.27 ± 8.4; *r* = 0.95 [0.94, 0.96], *p* ≪ 2.2e − 16). The CpG overlap between the five clocks with less than 1000 CpGs is presented in Fig. 2c, and the CpG overlap between all six clocks is presented in Additional file 1: Fig. S2. Since some of these clocks (Horvath, Hannum, and Li) were not based on the EPIC array, some CpGs were not included in our mAge calculations, as detailed in the methods section.

### The effect of the 18-month intervention on biological aging

Following 18 months of lifestyle interventions, and a relative weight loss of − 0.78 ± 4.9%, − 2.83 ± 5.3%, and − 3.9 ± 6.61% in HDG, MED, Green-MED, respectively, some aging clocks showed an increase or no change across all groups (e.g., Li mAge: 1.06 ± 1.98 years, 1.05 ± 1.97 years, 0.77 ± 2.98 years HDG vs. MED vs. Green-MED; *p* < 0.05 vs. baseline for all; Additional file 1: Fig. S3), while other clocks showed a significant increase or decrease in some but not all groups (e.g., PhenoAge and PCGrimAge). No significant differences between the intervention groups in both absolute and relative changes (18-month change accounting for baseline mAge) were observed for all clocks (Li mAge: *p* = 0.41 and *p* = 0.60 absolute and relative, respectively; Horvath mAge: *p* = 0.57 and *p* = 0.61; Horvath skin and blood mAge: *p* = 0.52 and *p* = 0.47; Hannum mAge: *p* = 0.67 and *p* = 0.57; PhenoAge: *p* = 0.34 and *p* = 0.69; PCGrimAge: *p* = 0.84 and *p* = 0.89). Among men only, similar results were observed (Li mAge: *p* = 0.39 absolute differences between the groups; Horvath mAge: *p* = 0.53; Horvath skin and blood mAge: *p* = 0.47; Hannum mAge: *p* = 0.64; PhenoAge: *p* = 0.14; PCGrimAge: *p* = 0.88; Additional file 1: Fig. S4).

Age acceleration and IEAA for all clocks, for the two time points (baseline vs. the end of the intervention) per intervention group, and between-group differences are presented in Additional file 1: Table S2. The aging pace DunedinPACE was reduced significantly within all intervention groups, but no significant differences between the groups were observed (*p* = 0.85).

### Green diet components positive effect on biological aging

**Fig. 3:**
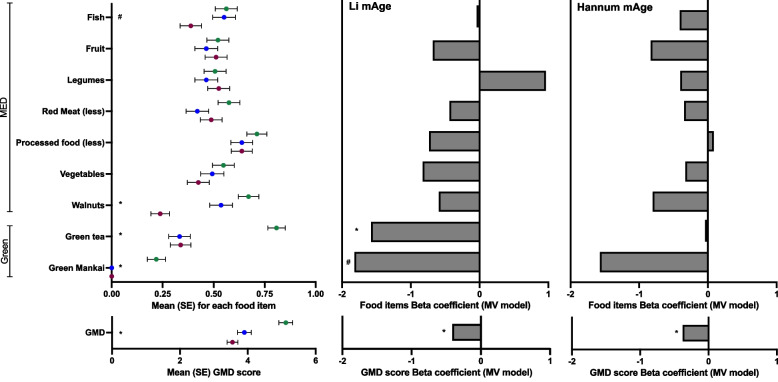
Green-MED adherence, components, and the association with mAge change. Left: forest plot of GMD adherence score (lower left) and its components (upper left) following 18 months of dietary intervention across groups with between-group differences indicated. Data presented as mean ± SE. Adherence was assessed using a 9-item score, ranging from 0 (non-adherence) to 9 (full adherence). Upper middle: association changes in the corresponding dietary component on the left with relative changes in Li mAge. Lower middle: the association of GMD score and relative change in Li mAge. Upper right: association changes in the corresponding dietary component on the left with relative changes in Hannum mAge. Lower middle: the association of GMD score and relative change in Hannum mAge. Data presented as beta coefficients; multi-variate models adjusted for age, sex, baseline mAge, and weight loss. Red dots = HDG; blue dots MED; green dots = green = MED. *p < 0.05; ^#^*p* < 0.1

We further examined the association between change in different clocks and the change in GMD score. Across all groups, the 18-month GMD component intake score was inversely associated with the relative change in Li mAge (beta =  − 0.338, *p* = 0.0178). In a multivariate (MV) model, the association between lower relative change in Li mAge remained significantly associated with the greater GMD score when controlling for age, sex, baseline mAge, and 18-month weight loss (beta =  − 0.41, *p* = 0.004; Fig. 3, lower middle panel). The distribution of the 9 components in the GMD score in each of the intervention groups is presented in Additional file 1: Additional file 1: Fig. S5. The Green-MED diet had the highest GMD score, followed by the MED and HDG (*p* = 1.149e − 06; Fig. 3, lower left panel). The intake of green Mankai, walnuts, and green tea (*p* = 2.467e − 08, *p* = 2.663e − 07, *p* = 1.358e − 06, respectively) differed between the intervention groups (Fig. 3, upper left panel), with fish intake showing marginal difference (*p* = 0.054). The green score components associated with lower mAge change were green Mankai and green tea intake (beta =  − 1.81 and *p* = 0.061, beta =  − 1.57 and *p* = 0.0016, respectively, multivariate model adjusted for age, sex, baseline Li mAge, and weight loss; Fig. 3, upper middle panel). The 18-month change in Li mAge acceleration and IEAA showed a similar pattern to the 18-month relative change in Li mAge, with an inverse association with the GMD score observed (Li mAge age acceleration: beta =  − 0.255, *p* = 0.004; Li mAge acceleration: beta =  − 0.288, *p* = 0.001; multivariate models).

Similar results for the associations of change in mAge and the 9-item GMD score were observed for the Hannum 18-month mAge relative change (beta =  − 0.377, *p* = 0.038; multivariate model; Fig. 3, lower right panel), Hannum age acceleration and IEAA changes (beta =  − 0.245, *p* = 0.04 and beta =  − 0.244, *p* = 0.04, respectively; multivariate models) but not for the Horvath 18-month relative mAge changes (beta =  − 0.222, *p* = 0.34), Horvath skin and blood (beta = 0.01, *p* = 0.40), PhenoAge (beta = 0.243, *p* = 0.52), PCGrimAge (beta = 0.013, *p* = 0.82), and DunedinPACE (beta = 0.001, *p* = 0.53).

### Epigenetic age and specific urine polyphenols

Lower 18-month relative change in Li mAge was associated with increased specific urine polyphenols, previously identified as related to a beneficial effect on different health outcomes in the DIRECT PLUS (the following urine polyphenol data were available for 249 (97.3%) of the participants with mAge measured): *hydroxytyrosol* (*r* =  − 0.185, *p* = 0.003), *urolithin C* (*r* =  − 0.158, *p* = 0.012), and *tyrosol* (*r* =  − 0.135, *p* = 0.03) (Additional file 1: Fig. S6a-c). A marginal correlation of relative change in mAge and *urolithin A* was observed (*r* =  − 0.11, *p* = 0.08). After further adjustment for age, sex, and weight loss, *tyrosol* remained the only urinary predictor for the 18-month relative change in Li mAge (beta =  − 1.828e − 06, corresponds to − 0.61 1SD change in *tyrosol*, *p* = 0.012). For the other epigenetic clocks, PCGrimAge and Horvath relative changes were correlated with *hydroxytyrosol* (*r* =  − 0.165, *p* = 0.008 and *r* =  − 0.158, *p* = 0.012, respectively) (Additional file 1: Fig. S6d-e), but the associations completely attenuated when examining the multivariate model (beta =  − 1.29e − 06, corresponds to -0.49 1SD change in *Hydroxytyrosol*, *p* = 0.219 for Horvath clock; beta = -7.96e-07, corresponds to − 0.07 1SD change in *hydroxytyrosol*, *p* = 0.432 for the PCGrimAge).

### Specific CpGs associated with the GMD score

**Fig. 4:**
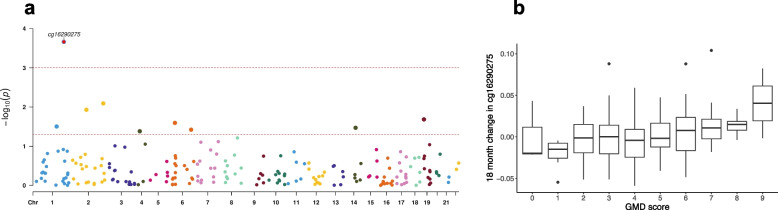
**a**, **b** Specific CpGs associated with the GMD score. **a** Correlations of the change in 217 CpGs from Li et al.’s mAge with GMD score. **b** Change in cg16290275 across GMD score. GMD score was assessed using a 9-item score, ranging from 0 (non-adherence) to 9 (full adherence). Boxplots represent the median, interquartile range, minimum, and maximum for the GMD score

As Li mAge showed the strongest association with GMD score, we further examined the 217 CpGs included in this mAge calculation. We calculated the 18-month difference in the CpGs (methylation at the end of the intervention − methylation at baseline) to correlate with the GMD score (Fig. 4a). Nine CpGs were correlated with GMD score (*p* < 0.05; Additional file 1: Table S3). Correction for multiple comparisons attenuated most of the associations—except for the change in cg16290275 (chr1:208,042,910, unannotated; *r* = 0.245, *p* = 2.17e − 04, FDR = 0.047; Fig. 4b).

### Subgroups analyses

**Fig. 5:**
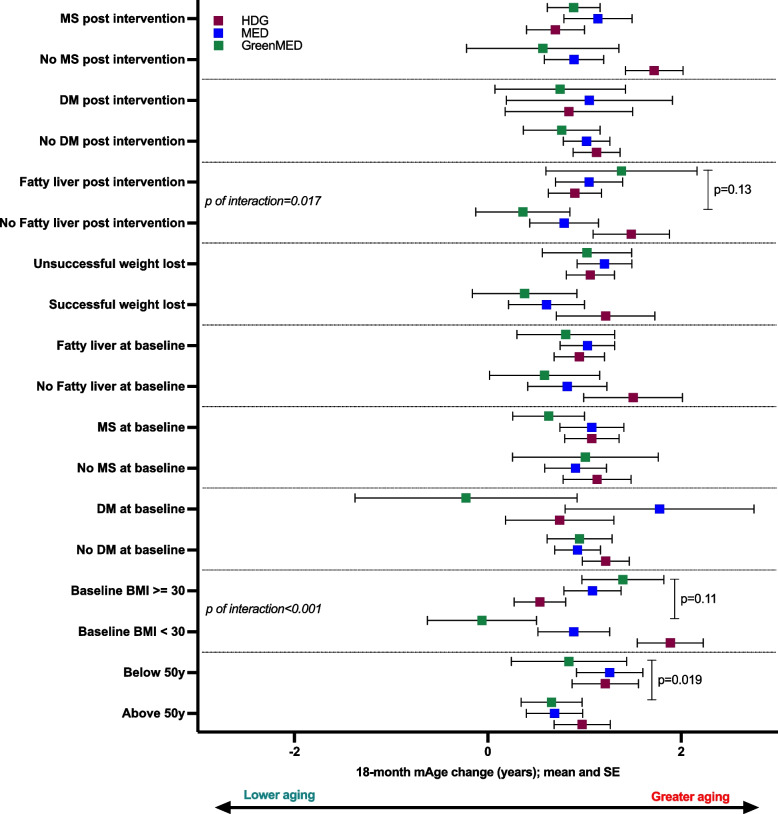
Biological aging across the intervention groups in subgroups of health status, men only. Forest plot showing the mean and SE of 18-month change in Li mAge. Data presented as mean and SE across health status and diet subgroups. The presence of DM was defined for participants with baseline fasting plasma glucose levels ≥ 126 mg/dL or hemoglobin-A1c levels ≥ 6.5% or if regularly treated with oral antihyperglycemic medications or exogenous insulin. Liver status was based on MRI-measured live fat, as published before [26], with a cutoff > 5% defining fatty liver. Interactions presented are between the health status and intervention. BMI, body mass index; DM, diabetes mellitus; MS, metabolic syndrome

Next, to quantify the magnitude of the effect size in the change in the Li mAge in subgroups, we examined specific subgroups of interest in a sex and intervention stratum. For this analysis, we used morbidity/risk data collected in the DIRECT PLUS: fatty liver, diabetes mellitus, metabolic syndrome, obesity, and age dichotomized at 50, as age 50 emerged as the most prominent threshold for changes in brain anatomy in our cohort [31]. Among men only (Fig. 5), Green-MED dieters above age 50 had the least Li mAge increase (i.e., more biological age attenuation) compared to those participants below 50 (0.66 ± 1.9 vs. 0.84 ± 3.8, 1.8%; beta = 2.1; *p* = 0.019, in a model, adjusted for 18-month weight change and baseline Li mAge), followed by the MED dieters (0.69 ± 1.7 vs. 1.26 ± 2.2; *p* = 0.17 adjusted model) and the HDG (0.98 ± 1.8 vs. 1.21 ± 2.11, *p* = 0.34, adjusted model). Non-obese participants at baseline and participants free of fatty liver disease after the intervention tended to benefit more from the Green-MED intervention (beta = 1.09, *p* = 0.11, and beta = 0.13, *p* = 0.13, respectively). We observed a significant interaction of improved liver status with the intervention group on Li mAge change (*p* = 0.017). The Green-MED diet was the main driver for the interaction, with *p* = 0.0165 for the Green-MED by liver status interaction term. Similarly, we observed a significant interaction of the intervention group by baseline obesity (*p* = 0.0006), with significant interactions for both MED diets (MED: *p* = 0.037; Green-MED: *p* = 0.00073). No age-by-intervention group interactions were observed (*p* = 0.81). For the presence of diabetes mellitus pre- and post-intervention (yes/no; defined for participants with baseline fasting plasma glucose levels ≥ 126 mg/dL or hemoglobin-A1c levels ≥ 6.5% or if regularly treated with oral antihyperglycemic medications or exogenous insulin) and metabolic syndrome score (below/above 3), no significant differences or interactions were observed. Among women only (Additional file 1: Table S4), we used univariate tests without further adjustments due to the small number of women in each group. No differences between the subgroups of health status were observed.

Finally, we examined the differences between the observed Li mAge at the end of the intervention and the expected mAge using the baseline linear regression of Li mAge ~ age, as previously demonstrated by us [16]. Overall, participants undergoing either MED-style diet had ~ 8.9 months difference between the observed Li mAge at the end of the intervention (64.95 ± 8.67 years) and the expected mAge (65.69 ± 7.91 years; *p* = 0.02). This was probably driven by the men undergoing one of the MED-style diets, as they had ~ 11.7 months difference between the observed and expected change in Li mAge (64.60 ± 8.31 years vs. 65.57 ± 7.82 years, *p* = 0.003). No differences between the observed and expected Li mAge were observed in the HDG group (*p* = 0.808).

## Discussion

In this randomized trial, we observed no reductions in biological aging among the three diet intervention groups. However, we found that beyond weight loss, adherence to a Green-MED diet regimen, especially with a greater intake of specific polyphenol-rich foods, might be associated with slower biological aging. In addition, mAge changes corresponded to the dynamic of specific urine polyphenols, previously identified as related to a beneficial effect on adiposity and brain anatomy outcomes in the DIRECT PLUS. To the best of our knowledge, this is the first clinical trial suggesting a specific link between polyphenol intakes and less biological aging.

Our study has some limitations. First, the GMD score is calculated based on self-reports and not on objective measurements. However, the dietary questionnaires used for this score were previously validated [28]. Second, the urine polyphenol analysis was qualitative in the form of intensities rather than concentrations, and we used values as relative to baseline change. In addition, the urine polyphenol assays were based on a spot urine sample rather than a 24-h urine collection, but these samples may have contained metabolites of polyphenols taken at the dinner. The small number of women limits our ability to generalize the results. The study population phenotype based on the inclusion criteria of abdominal obesity or dyslipidemia potentially differs heavily from the general population in other aspects than sex. Although we could not evaluate the cross-sectional associations of mAge signatures with the GMD score at baseline due to missing questions on processed meat, green Mankai, walnuts, and green tea, we could examine other specific food items. Nevertheless, this is the first large-scale, long-term study that reported the effect of polyphenol-rich foods on the attenuation of biological aging and the first to associate urine polyphenols with changes in mAge using both reported intakes used as a GMD score reflecting the green and polyphenol components and objective measurements of urine polyphenols. Another strength was the high 18-month retention rate of 89.8%, as previously reported [26].

Similar to previous studies, we observed a strong correlation between mAge and chronological age. It has to be noted that the chronological age was about 12 years lower than the mAge for both men and women. This may be due to several reasons, including lack of calibration, applying 207 CpGs out of 239 from the original Li mAge formula, for example, but may also reflect poor health status. Besides the association with age at baseline, we also assessed the cross-sectional association of mAge with dietary intake pre-intervention. Although weakly correlated, age and mAge by different clocks were significantly associated with fruit, vegetable, fish, and legume intake. However, we found no association between these foods and age acceleration. In previous studies, age acceleration was directly associated with fish, poultry, and fruit intake and inversely associated with alcohol across different mAge clocks [43, 44].

In this study, we could not find any between-group differences in mAge change between the interventions. Few intervention studies examined the differences in mAge changes between dietary intervention groups. An 8-week diet and lifestyle treatment led to a lower mAge change vs. a control group [15] in a sample of 43 healthy men. Another study of 219 women undergoing a 2-year diet and PA intervention [45] used epigenetic age acceleration measures according to the algorithm described by Lu et al. [46] to assess different interventions’ effects on biological aging. In contrast, two other intervention studies of either vitamin B12 and folic acid (24 months, 44 older men and women) or polyphenol supplementation (13 healthy male smokers) did not find significant between-group differences in mAge signatures [14]. A recent study [47] examining calorie restriction (25% below the participant’s baseline calorie intake) vs. control ad libitum diet for 2 years among 202 normal to overweight men and women did not find any significant differences between interventions in the change in multiple aging clocks from different generations. However, in their study, the aging pace “DunedinPACE” was slowed by the calorie restriction intervention after 12 months and maintained this pace through the follow-up at 24 months.

The combination of 9 dietary changes that included the intake of fruits, green tea, walnuts, green Mankai, fish, legumes, vegetables, and reduced red and processed meat was jointly associated with less biological aging and age acceleration. It has to be noted that we could detect associations between the change in two clocks (Li mAge and Hannum mAge) and GMD score, and not across all six epigenetic clocks estimated in this study. This finding, in addition to our report of favorable difference between the observed and expected Li mAge at the end of the intervention, should be interpreted with caution, as a number of measured and unmeasured confounders can explain these findings. We have tried to overcome this issue by using multivariate models adjusting for potential confounders such as age, sex, baseline mAge, and 18-month weight loss. However, the setting of the study does not allow any inference on a causal effect of polyphenols on biological age. Future studies should increase power by exploring the green diet pattern in a larger sample size, although it may be challenging to explore changes in epigenetic clocks since these require measuring DNA methylation at two time points in a setting of a clinical trial oriented to explore increased intake of polyphenols. Two observational studies have found an inverse association between mAge signatures and dietary scores of Dietary Approaches to Stop Hypertension diet (DASH), the Healthy Eating Index–2015, Alternative Healthy Eating Index (aHEI-2010), and the Alternative Mediterranean diet [48, 49]. A previous intervention study of 120 healthy elderly participants [12] found an inverse association of age acceleration with adherence to the Mediterranean-like diet using Horvath’s prediction models. In our study, increasing the intake of polyphenol-rich green tea and Mankai was associated with less biological aging. Polyphenols, secondary metabolites of plants with antioxidant properties [50], may have an anti-aging effect in several proposed mechanisms [51]: direct and indirect impact on nitric oxide synthase, modulation of miRNA expression related to longevity, and preventing cellular senescence. Considering polyphenols may also affect epigenetic modifications [18], the eating pattern of increasing polyphenols may also affect mAge, calculated based on DNA methylation levels. Thus, it may constitute an “epigenetic diet.”

The Li mAge was trained and tested on a set of 258 and 281 (respectively) Chinese and validated in two sets of 450 and 160 Chinese and Caucasians using whole blood methylation data [10]. The Li mAge was previously associated with exposure to polycyclic aromatic hydrocarbons in three panels of healthy Chinese participants [10]. In a sample of White participants with abdominal obesity or dyslipidemia, the Li age acceleration was associated with liver fat percentage and other cardiometabolic indicators [16]. Furthermore, the 18-month change in Li mAge was lower among people who improved their liver fat content and had successful weight loss. Searching for specific CpGs from the Li mAge formula associated with the GMD score to further examine the associations between polyphenols and epigenetics revealed that the change in cg16290275 was directly correlated with the GMD score. In several epigenome-wide association studies, whole blood cg16290275 methylation was associated with age and aging [52, 53]. This CpG site was also included in Hannum’s mAge model [35]. In children, cg16290275 methylation at birth was associated with increased BMI at 6 years old [54]. Our results further add information on specific foods and polyphenols associated with a lower increase in aging signatures and DNA methylation.

Lower methylation aging was associated with increased urolithins and tyrosols. Urolithins are the intestinal microbial metabolites from ellagic acid found in fruits, nuts, and seeds [55, 56]. *Urolithin A* and *urolithin C* were associated with reduced triglyceride accumulation and increased fatty acid oxidation in adipocytes and hepatocytes [57]. The phenylethanoids *tyrosol* and *hydroxytyrosol* originate in olives, green tea, and *Rhodiola* and may have an extensive effect on health due to their antiatherogenic, cardioprotective, anticancer, neuroprotective, and endocrine effects [58]. We selectively focused on these polyphenols following our previous work that identified increased *urolithin A* in the Green-MED group [21] and less so by the MED and HDG. Furthermore, increased *urolithin A* was associated with a reduction in visceral adiposity beyond age, sex, and waist circumference change [30]. Increased *urolithin A* and *tyrosol* were associated with lower age-related brain atrophy, represented by MRI-assessed hippocampal occupancy score [31]. The current work provides yet additional confirmation of the potential health benefits of consuming a high-polyphenol diet as the MED diet, specifically foods rich in urolithins and tyrosols.

The DIRECT PLUS trial included three lifestyle interventions: healthy dietary guidelines, the MED diet with the addition of 28 g/day of walnuts, and a Green-MED diet that included walnuts, green tea, and green Mankai shake while reducing the intake of red and processed meat. We showed previously that the Green-MED diet was effective in reducing liver (33) and visceral (28) fats and cardiometabolic risk (36) and is potentially neuroprotective for age-related brain atrophy (29). In a subgroup analysis, we observed that men above 50 benefited more in terms of aging attenuation following the Green-MED diet. This finding corresponds to previous work on age-related brain atrophy in older participants compared with younger participants. Atrophy was accelerated among those ≥ 50 years old, and brain atrophy was attenuated in groups receiving different-style MED diets, with the best outcomes among Green-MED diet participants [31]. Since the trajectory of some brain anatomy volumes varies nonlinearly by age [59], and there is an acceleration in the attenuation of the atrophy around the age of 55 years [60], providing nutritional strategy to attenuate mAge might have a significant role in healthy aging, beyond longevity. It has to be noted, however, that the effect of “regression to the mean” could occur when samples were selected based on extreme values in the population. This effect could be more profound for measures with inherited high variability, including large measurement error, such as MRI-assessed fat depots or brain anatomy in our study, but less for those that can be objectively and accurately measured, such as height and weight. Yet, this effect could occur to all subjects which were randomized into the intervention arms, so that the impact on the intervention effect would be minimal.

## Conclusions

While no specific lifestyle interventions showed greater benefit in terms of mAge attenuation, overall dietary adjustments reflected by the GMD score and urine polyphenols were inversely associated with biological aging, thus potentially contributing to longevity, although this should be further confirmed in complementary studies. Moreover, since these lifestyle changes were also associated with a beneficial effect on multiple health conditions, such as fatty liver, age-related brain atrophy, and cardiovascular risk, they might further contribute to healthy aging.

### Supplementary Information

**Additional file 1:** **Table S1.** Correlation of epigenetic clock measurements with the energy-adjusted fish, meat, fruit, legumes, and vegetable intakes. **Table S2.** Differences in mAge residuals (age acceleration) with/out controlling for cell type (Intrinsic epigenetic age acceleration). **Table S3.** Specific CpGs associated with the GMD score. Top correlations (*p*<0.05). **Table S4.** Biological aging across intervention groups in subgroups of health status, women only. **Fig. S1.** The residuals from a regression model of mAge on actual age. **Fig. S2.** Overlap between available CpGs for each clock. **Fig. S3.** 18-month absolute change in methylation age clocks across intervention groups for the entire cohort. **Fig. S4.** 18-month absolute change in methylation age clocks across intervention groups, men only. **Fig. S5.** The distribution of the 9 components in the GMD score in each intervention group. **Fig. S6.** Correlation between changes in different mAge clocks and specific urine polyphenols. 

## Data Availability

All data generated or analyzed during this study are included in this published article [and its supplementary information files]. Raw Illumina HumanMethylation850 Bead Chips Array data have been deposited in the ArrayExpress database at EMBL-EBI (www.ebi.ac.uk/arrayexpress [61]) with the accession number E-MTAB-12527 [32, 62]

## Abbreviations

BMI: Body mass index
CpG: Cytosine phosphate guanine
DNMT: DNA methyl transferase
FDR: False discovery rate
FFQ: Food Frequency Questionnaire
GEA: Gallic acid equivalents
GMD score: Green Mediterranean diet score
HDG: Healthy dietary guidelines
mAge: Methylation age
MED: Mediterranean
MRI: Magnetic resonance imaging
PA: Physical activity
WC: Waist circumference
