# Supplementary material for: The effect of polyphenols on DNA methylation-assessed biological age attenuation: the DIRECT PLUS randomized controlled trial
Source: BMC Med. 2023 Sep 25;21:364. doi: 10.1186/s12916-023-03067-3 (PMC10519069; doi:10.1186/s12916-023-03067-3)

The effect of polyphenols on DNA methylation-assessed biological age attenuation: The DIRECT PLUS trial

Anat Yaskolka Meir[1,2*], RD PhD; Maria Keller[3,4*], PhD; Anne Hoffmann[3] PhD; Ehud Rinott[1], MD PhD; Gal Tsaban[1,5], MD MPH; Alon Kaplan[1], MD PhD; Hila Zelicha[1], RD PhD; Tobias Hagemann[3], PhD; Uta Ceglarek[6], PhD; Berend Isermann[6], PhD; Ilan Shelef[5], MD; Matthias Blüher[3,4], MD; Michael Stumvoll[3,4], MD; Jun Li[7], MD PhD; Sven-Bastian Haange[8], PhD; Beatrice Engelmann[8], PhD, Ulrike Rolle-Kampczyk[8], PhD; Martin von Bergen[8,9], MD PhD; Frank B Hu[2,10,11], MD PhD; Meir J Stampfer[10,11], MD DrPH; Peter Kovacs[4#], PhD; Liming Liang[2#], PhD; Iris Shai[1,3,10#], RD PhD.

Additional file 1

Table of Contents

Table S1: Correlation of epigenetic clock measurements with the energy-adjusted fish, meat, fruit, legumes, and vegetable intakes. 2

Table S2: Differences in mAge residuals (age acceleration) with/out controlling for cell type (Intrinsic epigenetic age acceleration). 3

Table S3: Specific CpGs associated with the GMD score. Top correlations (p<0.05). 5

Table S4: Biological aging across intervention groups in subgroups of health status, women only 6

Figure S1: The residuals from a regression model of mAge on actual age 7

Figure S2: Overlap between available CpGs for each clock 8

Figure S3: 18-month absolute change in methylation age clocks across intervention groups for the entire cohort 9

Figure S4: 18-month absolute change in methylation age clocks across intervention groups, men only. 9

Figure S5: The distribution of the 9 components in the GMD score in each intervention group. 10

Figure S6: Correlation between changes in different mAge clocks and specific urine polyphenols. 11

Table S1: Correlation of epigenetic clock measurements with the energy-adjusted fish, meat, fruit, legumes, and vegetable intakes.

	Fish		Meat		Fruit		Vegetables		Legumes	
	r[1]	p	r[1]	p	r[1]	p	r[1]	p	r[1]	p
**Age**	0.158	**0.01**	0.003	0.96	0.284	**4.50E-06**	0.34	**3.20E-08**	0.264	**2.10E-05**
**Li mAge**	0.139	**0.03**	-7.80E-04	0.99	0.286	**3.90E-06**	0.32	**1.70E-07**	0.254	**4.60E-05**
**Li AA**	-0.012	0.84	0.019	0.76	0.093	0.137	0.01	0.852	0.04	0.52
**Li IEAA**	0.005	0.93	-0.089	0.16	0.043	0.492	0.07	0.205	0.046	0.46
**Horvath mAge**	0.167	**0.007**	0.019	0.76	0.293	**2.30E-06**	0.29	**1.80E-06**	0.262	**2.50E-05**
**Horvath AA**	0.055	0.37	0.041	0.52	0.092	0.14	-0.01	0.865	0.069	0.27
**Horvath IEAA**	0.056	0.37	0.009	0.88	0.077	0.220	-0.003	0.951	0.045	0.47
**Hannum mAge**	0.131	**0.04**	-0.002	0.96	0.279	**6.80E-06**	0.29	**2.70E-06**	0.237	**1.40E-04**
**Hannum AA**	0.004	0.95	-0.023	0.71	0.036	0.564	2.10E-05	0.999	0.037	0.55
**Hannum IEAA**	0.021	0.73	-0.061	0.33	0.009	0.880	0.04	0.539	0.045	0.47
**Horvath skin mAge**	0.164	**0.008**	0.0005	0.99	0.261	**2.71e-05**	0.325	**1.34e-07**	0.263	**2.30e-05**
**Horvath skin AA**	0.029	0.64	-0.019	0.75	-0.02	0.66	-0.005	0.93	0.031	0.61
**Horvath skin IEAA**	0.051	0.42	-0.053	0.39	-0.03	0.64	0.040	0.52	0.043	0.49
**PhenoAge**	0.136	**0.03**	0.003	0.96	0.251	**5.51e-05**	0.287	**3.68e-06**	0.217	**0.0005**
**PhenoAge AA**	0.005	0.93	0.007	0.90	-0.023	0.66	-0.022	0.71	-0.003	0.96
**PhenoAge IEAA**	0.016	0.79	-0.0002	0.99	-0.037	0.55	-0.007	0.91	-0.011	0.85
**GrimAge**	0.151	**0.02**	0.025	0.69	0.233	**0.0001**	0.273	**1.09e-05**	0.239	**0.0001**
**Grim AA**	0.053	0.40	0.099	0.11	-0.082	0.19	-0.109	0.08	-0.011	0.86
**Grim IEAA**	0.033	0.59	0.099	0.11	-0.09	0.12	-0.141	**0.024**	-0.019	0.75
**DunedinPACE**	0.021	0.73	0.017	0.78	-0.003	0.96	0.046	0.46	0.062	0.32

[1]Spearman correlation coefficient. AA, age acceleration; IEAA, intrinsic epigenetics age acceleration; mAge, methylation age.

Table S2: Differences in mAge residuals (age acceleration) with/out controlling for cell type (Intrinsic epigenetic age acceleration).

a. Age acceleration

	Age acceleration pre intervention (baseline)	Age acceleration post intervention	p-value for paired t-test	p between groups baseline	p between group post intervention
**Li AA**				0.577	0.563
HDG	0.32 (3.7)	0.43 (3.7)	0.573		
MED	-0.46 (4.4)	-0.36 (4.4)	0.891		
Green-MED	0.104 (4.2)	-0.097 (3.9)	0.519		
**Horvath AA**				0.144	0.296
HDG	0.65 (5.0)	0.49 (5.1)	0.752		
MED	-0.32 (4/2)	-0.31 (4.1)	0.973		
Green-MED	-0.37 (4.5)	-0.21 (4.1)	0.403		
**Hannum AA**				0.444	0.272
HDG	0.49 (4.9)	0.63 (4.8)	0.671		
MED	-0.45 (4.6)	-0.51 (4.9)	0.865		
Green-MED	-0.08 (5.4)	-0.17 (4.9)	0.809		
**Horvath skin and blood AA**				0.16	0.068
HDG	0.67 (3.5)	0.84 (3.7)	0.485		
MED	-0.57 (4.2)	-0.65 (4.3)	0.740		
Green-MED	-0.15 (3.9)	-0.25 (3.8)	0.725		
**PhenoAge AA**				0.677	0.217
HDG	0.59 (4.4)	0.96 (5.2)	0.401		
MED	-0.92 (5.6)	-1.04 (5.7)	0.770		
Green-MED	0.25 (6.2)	0.0001 (4.5)	0.649		
**PCGrimAge AA**				0.373	0.416
HDG	0.29 (2.9)	0.24 (3.0)	0.698		
MED	-0.25 (2.2)	-0.19 (2.2)	0.508		
Green-MED	-0.05 (2.4)	-0.07 (2.4)	0.911		
**DunedinPACE**				0.672	0.587
HDG	1.12 (0.1)	1.11 (0.1)	0.022		
MED	1.10 (0.1)	1.08 (0.1)	0.058		
Green-MED	1.1 (0.1)	1.1 (0.1)	0.036		

Data presented as mean (SD). AA, age acceleration; HDG, healthy dietary guidelines; MED, Mediterranean.

b. Intrinsic epigenetic age acceleration

	Age acceleration pre intervention (baseline)	Age acceleration post intervention	p-value for paired t-test	p between groups baseline	p between group post intervention
**Li IEAA**				0.461	0.247
HDG	0.41 (3.3)	0.46 (3.1)	0.793		
MED	-0.43 (4.1)	-0.32 (4.2)	0.613		

Green-MED	-0.007 (3.8)	-0.16 (3.6)	0.634		
**Horvath IEAA**				0.0392	0.102
HDG	0.85 (4.9)	0.63 (4.8)	0.673		
MED	-0.36 (4.0)	-0.26 (3.9)	0.754		
Green-MED	-0.51 (4.1)	-0.39 (3.7)	0.747		
**Hannum IEAA**				0.2	0.152
HDG	0.65 (4.6)	0.70 (4.1)	0.893		
MED	-0.49 (4.0)	-0.53 (4.3)	0.923		
Green-MED	-0.20 (4.6)	-0.21 (4.3)	0.970		
**Horvath skin and blood IEAA**				0.076	0.031
HDG	0.77 (3.4)	0.91 (3.1)	0.575		
MED	-0.63 (3.9)	-0.68 (4.0)	0.812		
Green-MED	-0.21 (3.6)	-0.28 (3.7)	0.781		
**PhenoAge IEAA**				0.58	0.16
HDG	0.66 (4.1)	1.06 (4.9)	0.323		
MED	-0.96 (5.6)	-1.16 (5.5)	0.632		
Green-MED	0.22 (5.9)	0.008 (4.3)	0.693		
**PCGrimAge AA**				0.382	0.355
HDG	0.25 (2.6)	0.27 (2.6)	0.725		
MED	-0.21 (2.0)	-0.22 (2.1)	0.727		
Green-MED	-0.05 (2.2)	-0.06 (2.2)	0.976		

Data presented as mean (SD). HDG, healthy dietary guidelines; IEAA, intrinsic epigenetic age acceleration; MED, Mediterranean.

Table S3: Specific CpGs associated with the GMD score. Top correlations (p<0.05).

cgID	r (Spearman)	p-value	FDR
**Δcg16290275**	0.24576	**0.000217**	**0.047164**
**Δcg03172765**	-0.17728	0.008108	0.853361
**Δcg22454769**	-0.16875	0.011798	0.853361
**Δcg03032253**	-0.15539	0.02054	0.992848
**Δcg13187764**	-0.15017	0.025251	0.992848
**Δcg13576006**	0.144667	0.031188	0.992848
**Δcg07481335**	0.142615	0.033688	0.992848
**Δcg08202165**	0.13959	0.037683	0.992848
**Δcg09354241**	-0.13708	0.04129	0.992848

FDR, false discovery rate.

Table S4: Biological aging across intervention groups in subgroups of health status, women only.

	HDG N=10				MED N=9				Green-MED N=9			
	N	Mean mAge[1]	SE	p-value[2]	N	Mean mAge[1]	SE	p-value[2]	N	Mean mAge[1]	SE	p-value[2]
**Above 50y**	3	-0.44	3.6	0.49	3	0.66	1.7	0.70	6	0.48	1.5	0.52
**Below 50y**	7	1.33	1.3		6	1.83	2.3		2	1.41	2.2	
**Baseline BMI < 30**	5	-0.12	2.9	0.40	3	1.19	2.6	0.89	4	0.21	2.5	0.39
**Baseline BMI >= 30**	5	1.71	0.47		6	1.56	3.7		5	1.25	0.68	
**No DM at baseline**	10	0.79	2.2	NA	7	1.42	2.4	0.88	9	0.79	1.7	NA
**DM at baseline**	-	-	-		2	1.49	0.2		-	-	-	
**No MS at baseline**	9	0.74	2.3	NA	3	0.41	0.70	0.15	7	0.28	1.5	0.11
**MS at baseline**	1	-	-		6	1.95	2.4		2	2.55	1.2	
**No Fatty liver at baseline[3]**	7	0.51	2.6	1	4	0.36	1.3	0.19	6	0.65	1.6	0.62
**Fatty liver at baseline[3]**	3	1.46	0.3		4	2.45	2.7		2	2.03	2.0	
**Successful weight lost**	0	-	-	NA	2	0.98	1.5	1	2	1.89	2.5	0.19
**Unsuccessful weight lost**	10	0.79	2.2		7	1.57	2.3		7	0.47	1.8	
**No Fatty liver post intervention[4]**	5	0.09	3.0	0.71	5	0.31	1.1	NA	6	0.16	1.5	NA
**Fatty liver post intervention[4]**	4	1.7	0.5		1	-	-		1	-	-	
**No DM post-intervention**	10	0.79	2.2	NA	7	1.42	2.4	0.88	9	0.79	1.7	NA
**DM post-intervention**	-	-	-		2	1.49	0.17		-	-	-	
**No MS post-intervention**	9	0.74	2.3	NA	5	0.49	1.4	0.39	7	0.28	1.5	0.11
**MS post-intervention**	1	-	-		4	2.61	2.4		2	2.55	1.2	

[1]mAge presented as absolute change. [2]Mann-Whiney test. [3] Liver MRI available for 26 participants at baseline. [4] Liver MRI available for 22 participants at T18. The presence of DM was defined for participants with baseline fasting plasma glucose levels ≥126 mg/dL or haemoglobin-A1c levels ≥6.5% or if regularly treated with oral antihyperglycemic medications or exogenous insulin. Liver status was based on MRI-measured live fat, as published before [35], with a cutoff >5% defining fatty liver. Interactions presented are between the health status and intervention group. BMI, body mass index; DM, diabetes mellitus; HDG, healthy dietary guidelines; MED, Mediterranean; MS, metabolic syndrome.

Figure S1: The residuals from a regression model of mAge on actual age. Positive residuals (red) represent mAge greater than age (biologically older). Negative residuals (green) represent mAge smaller than age (biologically younger).

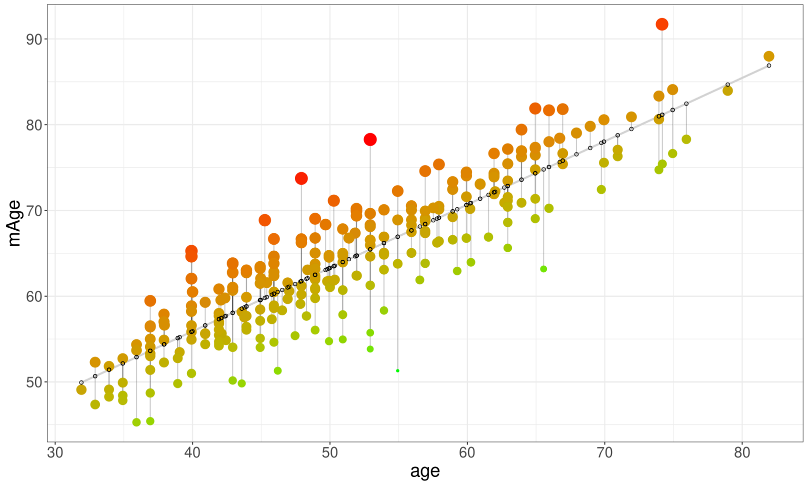

Figure S2: Overlap between available CpGs for each clock

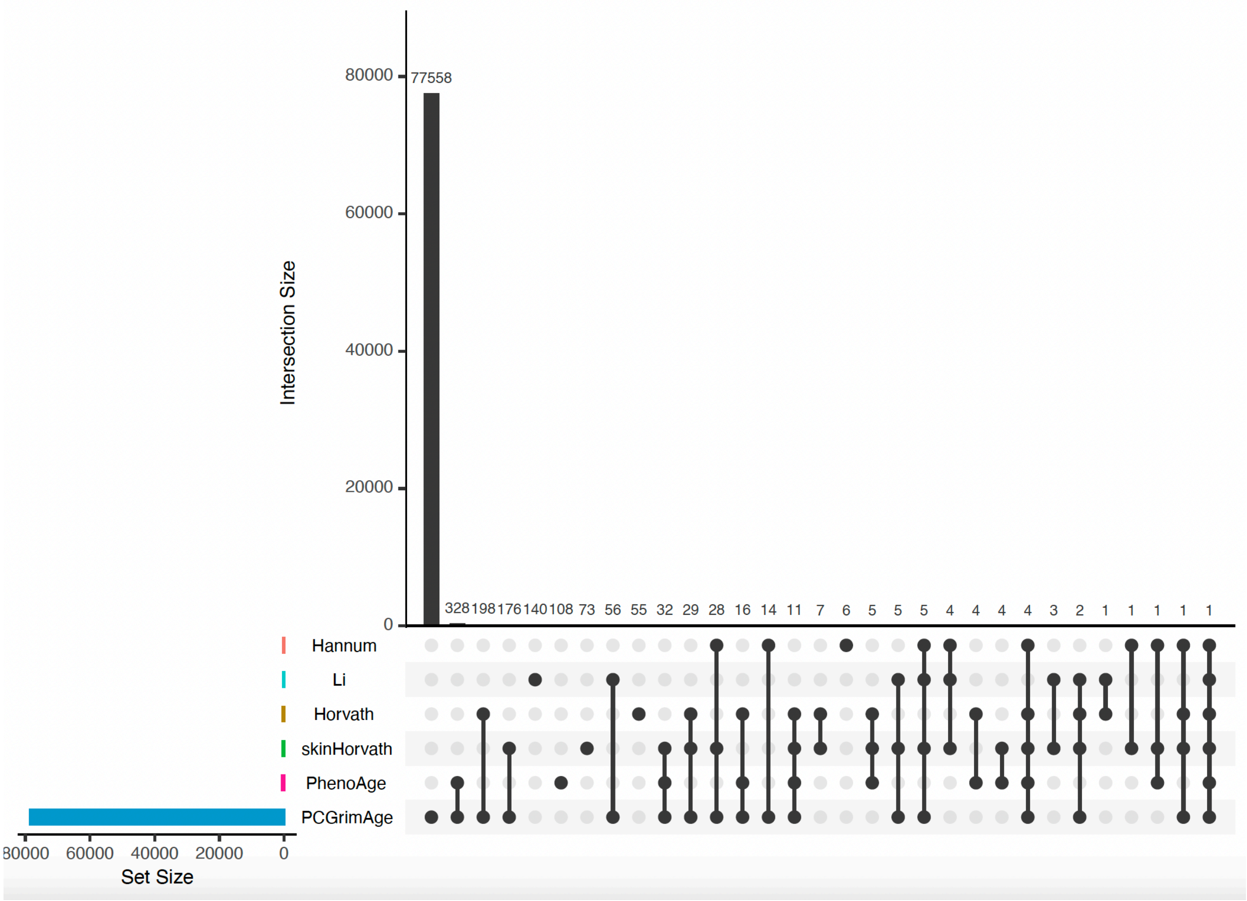

Figure S3: 18-month absolute change in methylation age clocks across intervention groups for the entire cohort. (a) Li mAge; (b) Horvath mAge; (c) Horvath skin and blood mAge; (d) Hannum mAge; (e) PhenoAge; (f) PCGrimAge. * $p<0.05$ vs. baseline.

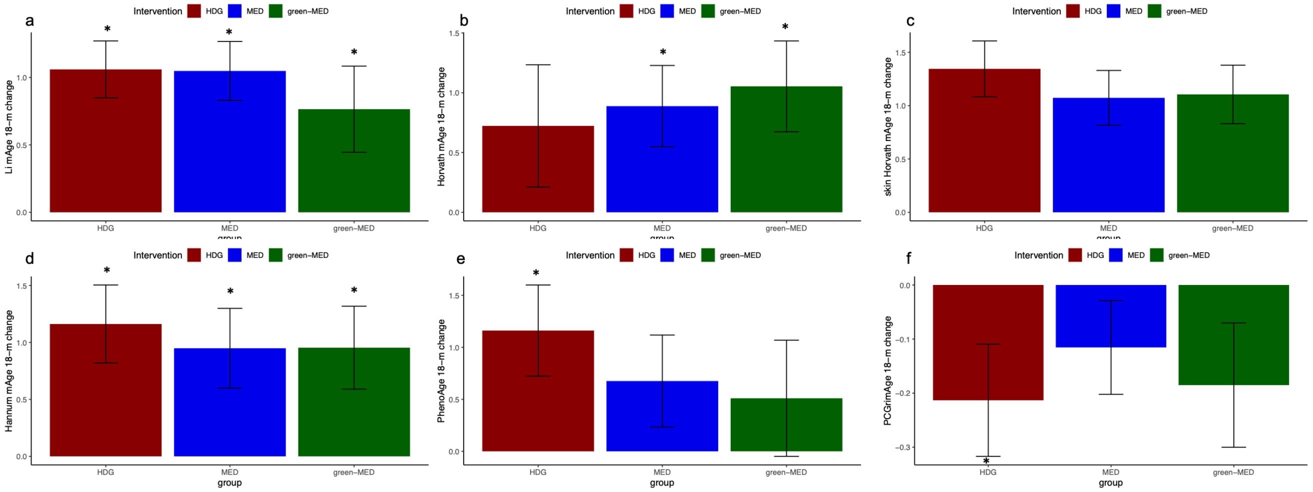

Figure S4: 18-month absolute change in methylation age clocks across intervention groups, men only. (a) Li mAge; (b) Horvath mAge; (c) Horvath skin and blood mAge; (d) Hannum mAge; (e) PhenoAge; (f) PCGrimAge.

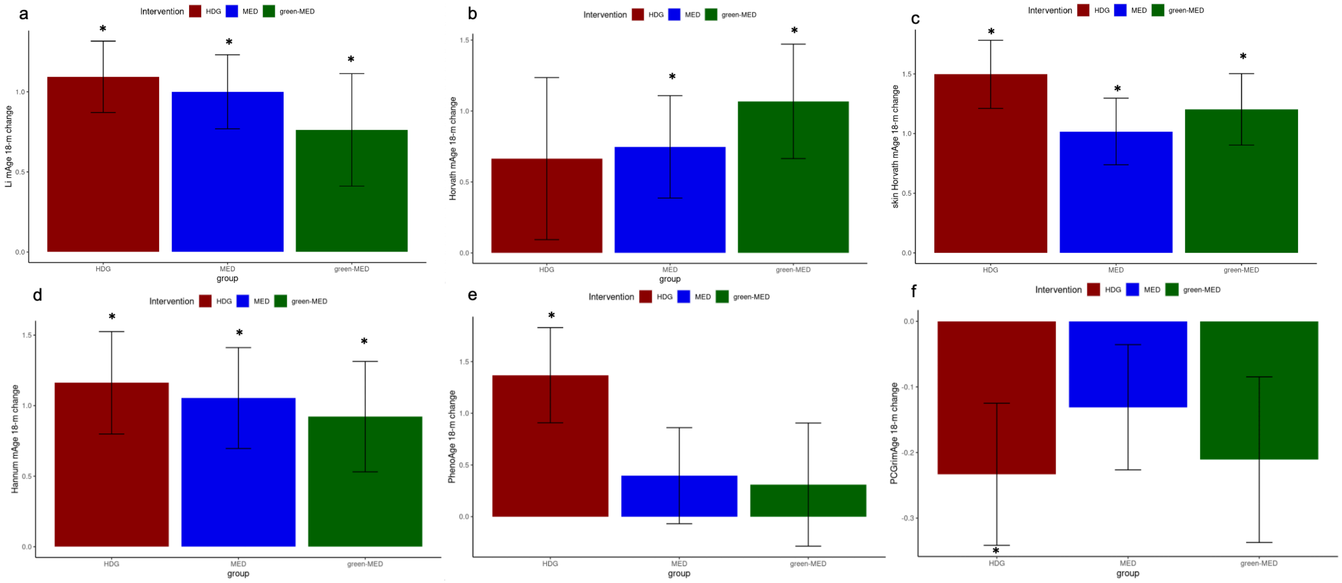

Figure S5: The distribution of the 9 components in the GMD score in each intervention group. Preferred below median: Fruit, vegetables, legumes, fish, green tea, walnuts, and green Mankai. preferred above median: Meat and processed food. a. Healthy dietary guidelines; b. Mediterranean diet supplemented with walnuts; c. Green Mediterranean low in red and processed meat and supplemented with walnuts, green tea, and green Mankai.

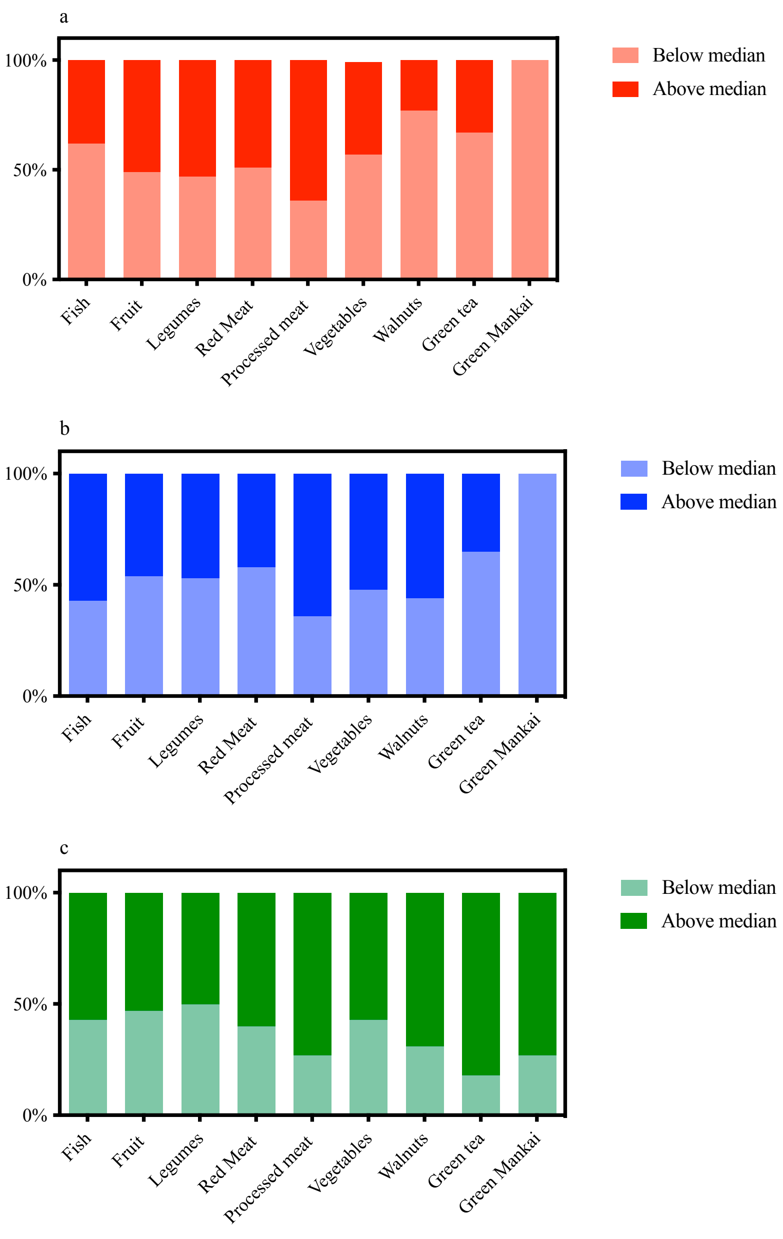

Figure S6: Correlation between changes in different mAge clocks and specific urine polyphenols. a. The correlation between 18-month relative change in Li mAge and Hydroxytyrosol; b. The correlation between 18-month relative change in Li mAge and Tyrosol; c. The correlation between 18-month relative change in Li mAge and Urolithin C; d. The correlation between 18-month relative change in Horvath mAge and Hydroxytyrosol; e. The correlation between 18-month relative change in PCGrimAge mAge and Hydroxytyrosol. Blue line is a linear curve; red dashed line is a smooth curve.

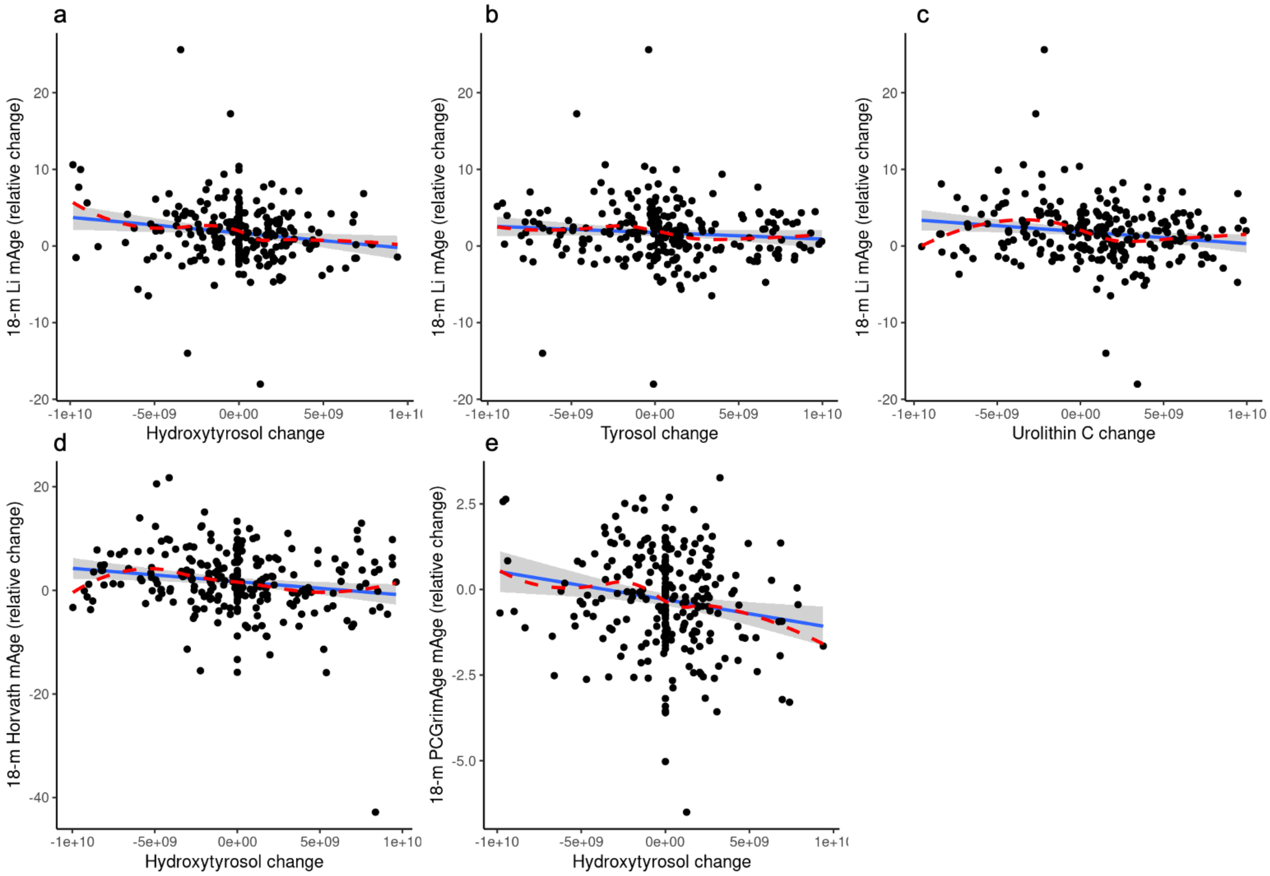

Supplement: Supplementary file 1 — Additional file 1: Table S1. Correlation of epigenetic clock measurements with the energy-adjusted fish, meat, fruit, legumes, and vegetable intakes. Table S2. Differences in mAge residuals (age acceleration) with/out controlling for cell type (Intrinsic epigenetic age acceleration). Table S3. Specific CpGs associated with the GMD score. Top correlations (p<0.05). Table S4. Biological aging across intervention groups in subgroups of health status, women only. Fig. S1. The residuals from a regression model of mAge on actual age. Fig. S2. Overlap between available CpGs for each clock. Fig. S3. 18-month absolute change in methylation age clocks across intervention groups for the entire cohort. Fig. S4. 18-month absolute change in methylation age clocks across intervention groups, men only. Fig. S5. The distribution of the 9 components in the GMD score in each intervention group. Fig. S6. Correlation between changes in different mAge clocks and specific urine polyphenols. [file 12916_2023_3067_MOESM1_ESM.pdf]
